# Physicochemical Parameters and Alarming Coliform Count of the Potable Water of Eastern Himalayan State Sikkim: An Indication of Severe Fecal Contamination and Immediate Health Risk

**DOI:** 10.3389/fpubh.2019.00174

**Published:** 2019-07-10

**Authors:** Ashish Kumar Singh, Saurav Das, Samer Singh, Nilu Pradhan, Varsha Rani Gajamer, Santosh Kumar, Yangchen D. Lepcha, Hare K. Tiwari

**Affiliations:** ^1^Department of Microbiology, School of Life Sciences, Sikkim University, Gangtok, India; ^2^Department of Microbial Biotechnology, Panjab University, Chandigarh, India; ^3^Centre of Experimental Medicine & Surgery, Institute of Medical Sciences, Banaras Hindu University, Varanasi, India; ^4^State Institute of Rural Development, Government of Sikkim, Gangtok, India

**Keywords:** Sikkim springs, community reservoir, household water, physicochemical parameters, *E. coli*, *Enterococcus*, total coliform, correlation coefficient

## Abstract

Continuous decline in potable water sources has raised serious concerns over human health. Developing countries are the most affected in this regard due to a lack of proper hygiene maintenance. Sikkim, an Eastern Himalayan state with mountains as the predominant topological features, harbors several perennial natural springs. Spring water is the primary source of potable water for the population in four districts of the state viz. East, West, North and South. Recent outbreaks of water-borne diseases and the relative lack of scientific studies on its potential correlation with the water quality of the area have educed this study. Physicochemical parameters of springs, community reservoirs, and household water were analyzed by ICP-MS and multi probe meter. Using the membrane filtration method, the microbial quality of the water samples during different seasons was assessed, primarily evaluating the presence of fecal indicators viz. *Escherichia coli*, total coliform and *Enterococcus*. The seasonal risk category of the water sources was also determined. Most of the physicochemical parameters of the spring water were within the permissible limits of WHO standards. However, water from four districts was recorded with traces of toxic heavy metals like mercury (0.001–0.007 mg/l), lead (0.001–0.007 mg/l), and selenium (0.526–0.644 mg/l), which are above the permissible limits of WHO. All the spring water samples were categorized as Mg-HC${\text{O}}_{3}^{-}$ type and can be predicted as shallow fresh ground water based on the piper analysis. Microbial confirmatory testing indicated severe fecal contamination of water sources with high counts of total coliform (TC), *Escherichia coli* (EC) and *Enterococcus* (EN). The highest level of TC was recorded from West Sikkim (37.26 cfu/100 ml) and the lowest in North Sikkim (22.13 cfu/100 ml). The highest level of contamination of *E. coli* and *Enterococcus* was found in East Sikkim (EC = 8.7 cfu/100 ml; EN = 2.08 cfu/100 ml) followed by South Sikkim (EC = 8.4 cfu/100 ml; EN = 2.05 cfu/100 ml). There was a significant positive correlation between the contamination levels of the spring water and the community reservoir tank. As far as the seasonal variation is concerned, the rainy season showed the most contamination with coliform correlating with a high incidence of different water-borne diseases (East = 86%; West = 100%; South = 100%; North = 80%).

## Introduction

Access to safe drinking water is critical to human health and development (1). Urbanization and increasing anthropogenic activity have wedged the Earth's natural environment and negatively impacted human health (2). Contamination of water sources has become a cause of concern as it is directly related to the health index of people. Around the world, water contamination causes death from gastrointestinal diseases like diarrhea, dysentery, cholera, and typhoid on a regular basis. Developing countries are particularly worse affected due to insufficient availability of potable water and lack of good health care systems (3). The increasing incidence of antibiotic resistance among water-borne pathogens is also aggravating the problem (4). Antibiotic resistant bacterial inhabitants of fresh water can colonize and become incorporated in water distribution systems and create widespread epidemics (5, 6). Three main elements that significantly affect the quality of drinking water in water distribution network are the quality of raw water at the source, the purification process employed for water and the distribution system used for water (7). These three elements affect the physicochemical characteristics as well as the microbial composition of drinking water (8). Several microbial communities survive in the water system, which in a favorable environment start multiplying in the water and depreciate the water quality and cause serious health problems (7). Microbial contamination of drinking water remains a persistent serious problem in developing countries, including India. Microbial contamination of water sources is primarily contributed by human or animal fecal materials. In India, sewage often finds its way into city drains and finally into the rivers, contributing to the poor quality of water sourced from the river. Additionally, due to unavailability of proper fecal sludge treatment, most of the sewage and fecal sludge find their way to the ground and contribute toward the bacteriological contamination of groundwater (9). A survey report from Lancet in 2014 projected that about 24% of children in the urban areas and 55% in the rural areas of India were falling ill due to contaminated water (10). Furthermore, about 11% of urban households and 23% of rural households suffered an infant death due to consumption of contaminated water (10). As a result of the poor quality of drinking water, diarrhea has become the fourth leading cause of death worldwide. In 2015 alone it caused an estimated 1.3 million deaths of children under the age of 5 years (11). Together, these facts warrant research on drinking water quality with respect to hydrochemistry and microbiology to ascertain the associated health risk for the residents of any area.

Water quality is determined by both hydrochemistry and microbiological profile. Geochemical processes like weathering, dissolution, hydrolysis, precipitation, adsorption, and ion exchange as well as oxidation reduction and biochemical reactions are major controlling factors for the chemistry of groundwater (12). Assessment of physicochemical parameters like pH, electrical conductivity (EC), total dissolved solids (TDS), alkalinity, and the levels of fluoride, arsenic, lead, and nitrate is generally considered to set guidelines and categorize the physicochemical water quality. In microbiological analysis, determination of indicator bacteria like *Escherichia coli*, total coliform and *Enterococcus* are generally performed to assess the possibility of fecal contamination and to qualify the source for potable use (13).

Increasing population with diminishing potable water sources is the biggest concern of the Twenty-first century when it comes to meeting the demand of hygienic water. The present global population is around 7.6 billion and growing at a rate of 83 million per year, globally (14). Surface water and groundwater act as the major drinking water sources for humans and other organisms (15). Groundwater is considered a reliable source of fresh water which is easy to access for various purposes such as domestic, industrial, irrigation, etc. (16). Worldwide, ~1.5 billion people are directly or indirectly dependent on groundwater for their domestic and agricultural needs (17). Over half (56%) of rural Indian people have access to potable water from tube wells, while 14% from open wells and 25% by supplied water system (16). However, industrial (toxic element), agricultural (runoff fertilizers), and microbial contamination of groundwater sources is the major concern. Reports from the WaterAid mentioned that even households that receive drinking water from “improved” sources (42% in urban and 60% in rural) are also above the permissible limits of WHO (9).

Sikkim is an Eastern Himalayan state of India and lies between the South East Asian countries Nepal and Bhutan. The state is characterized by a tremendous scarcity of potable and treated drinking water for the rural as well as urban population. The crisis is much more severe in the urban and city areas relatively, because of a deficiency of fresh water sources and water contamination. The Himalayan range is a source of countless perennial springs, and the mountain people depend largely on spring water for their sustenance. The mountain springs, locally known as “*Dharas*,” are the natural discharges of groundwater from various aquifers. Some of the springs which are considered sacred, are revered as “*Devithans*” and are protected from biotic interferences (18). In rural areas of Sikkim, the majority of potable water supplied for drinking and domestic purpose is sourced from spring water without proper filtration and treatment. As per an estimate, about 80% of the total rural community is solely dependent on spring water for their domestic consumption and drinking purposes (18). The dependence of the people of the state on spring water underlines the importance of the current study. This study for the first time reports the quality of springs, community reservoirs and household water from four districts—East, West, South and North—in terms of both physicochemical and microbiological parameters, a qualitative as well as a quantitative index. The data presented here could help structure and lay out the important foundation for future water treatment protocols for the region by the government agencies and also formulate policies to tackle the immediate health risk. Similar work in the regions of the world where similar conditions prevail (e.g., South East Asia) could also help improve the overall health of the local population and provide a roadmap to advise a cohesive, comprehensive, and inclusive development plans.

## Materials and Methods

### Description of Study Site

**Figure 1:**
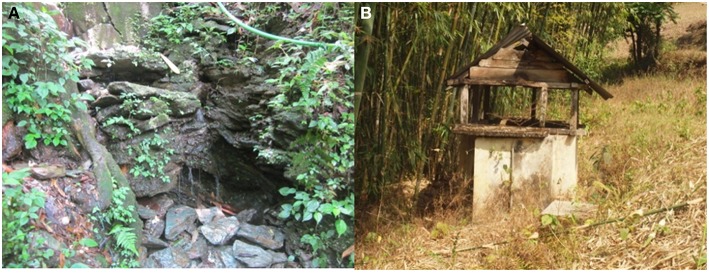
Photograph of the study areas: **(A)** spring water **(B)** a typical community reservoir where water from the springs was stored before being distributed to the households.

**Figure 2:**
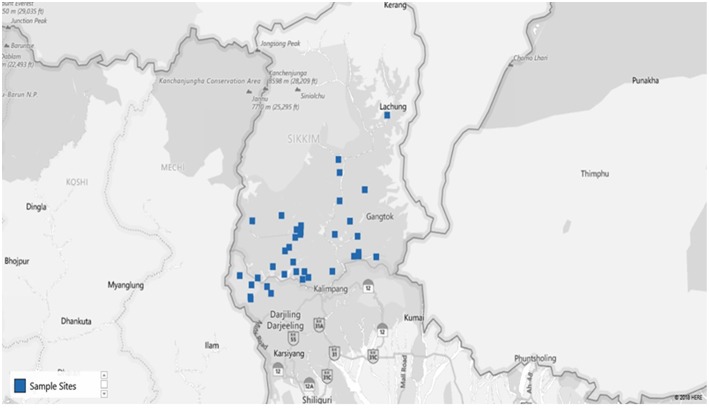
Map of the study site (blue dots specify the villages from where samples were collected). [Map chart was prepared in Microsoft Excel (office 365)] (Supplementary Table S1).

The study was conducted in different villages of Sikkim, which is a North Eastern state of India. The state is located off the slopes of the Eastern Himalayas at a latitude and longitude of 27°31′58″ N, 088°30′43″ E, covering ~115 km from North to South and 65 km from East to West. The landscape of this area varies, with an altitude between 300 and 8,583 meters above sea level comprising lower, middle and higher hills, alpine zones, and snowbound land. Sikkim is a multi-ethnic state in which the population is divided into tribal and non-tribal groups (18). Most of Sikkim's population is dependent on spring water for their potable use (80%) which is locally known as “Dharas” (Figure 1A). Among these springs, many are considered blessed, are known as “Devithans” and are safeguarded from any biotic interferences (18). There is a three-phase supply chain in the state. Spring water is collected at the storage tank (community reservoirs) built by the Rural Management and Development Department (RMDD), Government of Sikkim at the community and village level (Figure 1B). The collected water is then distributed to households for domestic purposes and to some extent for the irrigation of kitchen gardens as well as greenhouse crops (Figure 2) (18).

### Study Design

**Figure 3:**
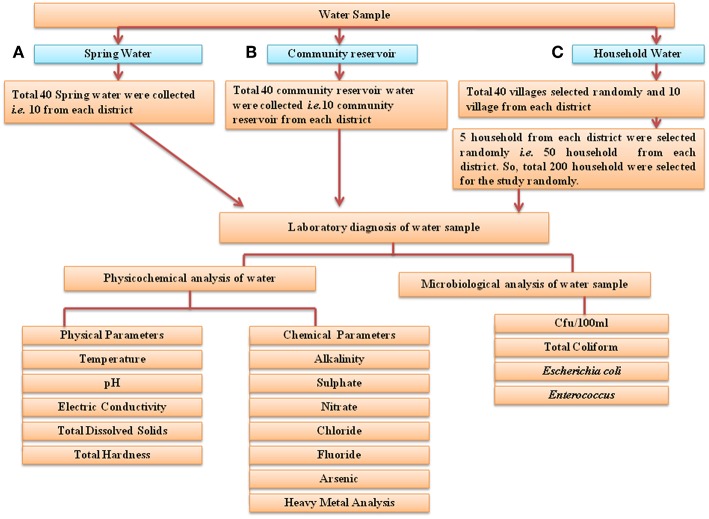
Flowchart of the study design. Three different types of water samples were collected: **(A)** spring water, **(B)** community reservoir water, and **(C)** household water. They were evaluated for microbial and physicochemical analysis as indicated. In total, water from 40 springs—i.e., from 10 springs from each district, water from 40 community reservoirs and 200 household water samples (50 household samples from each of the four districts) was collected for this study.

For this study, 40 springs that served as potable water sources and 40 community reservoirs were selected (10 from each district: East, West, South, and North) (Figure 3). For household water samples, 10 villages were selected from each district, with a total of 40 villages, and water samples from 5 households of each village were collected (10 × 4 × 5 = 200 samples). Springs were selected based on the altitude, minimum number of households dependent on a spring and perennial nature (19) (Supplementary Table S2). Different Gram Panchayat Units (GPUs) or village level blocks were selected for the current study on the basis of recent reports of water-borne disease incidence (6). At each household, the respondent was requested to provide water which they were using for drinking. A structured questionnaire was used for the survey (Supplementary Table S3).

### Sampling Procedure

Water samples were collected in 1 L sterile, wide mouth, plastic bottles (Nalgene, USA). Before collection, containers were sterilized using autoclave and washed with aqueous sodium thiosulfate solution [100 g/l (w/v)]. While collecting the sample, the bottles were completely submerged into water and were opened inside the water source to avoid air contamination. The container was filled by holding it diagonally submerged. A gap of 2 cm was left between the cap and water to provide sufficient airspace for mixing the water before analysis (20).

### Physicochemical Analysis

Physicochemical parameters of spring water like temperature (°C), pH, electrical conductivity (EC) (μS/cm) and TDS (mg/l) were measured by a multi-probe potable meter (Hi-media, Mumbai, India). Alkalinity (ppm), total hardness (TH) (mg/l), levels of sulfate (mg/l), nitrate (mg/l), chloride (mg/l), and fluoride (mg/l) were measured by an Aqua check water testing kit provided by Hi-media, Mumbai, India. Elemental detection was performed by ICP-MS (Inductive Coupled-Plasma Mass Spectroscopy) (Perkin-Elmer NexIon 300X, USA). The assessed elements were indium (In), barium (Ba), lead (Pb), silver (Ag), aluminum (Al), arsenic (As), barium (Ba^−1^), beryllium (Be), bismuth (Bi), calcium (Ca), cadmium (Cd), cobalt (Co), chromium (Cr), cesium (Cs), copper (Cu), iron (Fe), gallium (Ga), sulfur (S), potassium (K), lithium (Li), magnesium (Mg), manganese (Mn), sodium (Na), nickel (Ni), molybdenum (Mo), rubidium (R), selenium (Se), strontium (Sr), titanium (Ti), uranium (U), boron (B), vanadium (V), zinc (Zn), mercury (Hg), silicon (Si), phosphorus (P), nitrogen (N), chlorine (Cl), zirconium (Zr), xenon (Xe), tin (Sn), iodine (I), and cerium (Ce). All the water samples were collected and analyzed directly without any pretreatment except for arsenic and nitrate. Water samples for analysis of arsenic and nitrate were mixed with the acid (1 M HCl, 5 ml for 500 ml water sample) (21).

### Microbiological Analysis

For microbiological analysis, water samples were collected separately in 1 L sterile containers. Samples for microbiological analysis were transported in ice-cooled conditions. Water samples were collected in triplicate. Membrane filtration was used for the detection of *E. coli* and total coliform counts (22). For the detection of total coliform, the membrane filter was placed on M-Endo agar LES (Hi-Media) and the bacterial colonies developed were counted by a colony counter (Cole-Parmer, India). For the detection of *E. coli*, membrane filters were cultured on Rapid Hi-chrome Agar (Hi-media, M1465). All plates were incubated at 35 ± 2°C for 24 h. The positive bacterial isolates of indole test based on blue green fluorescence under UV-light exposure were identified as *E. coli*. For the detection of the *Enterococcus* group, the filter was incubated on m-*Enterococcus* agar at 41°C for 48 h (23). The density of the coliform and fecal streptococci was calculated using the following equation as proposed by American Public Health Association (23).

Total (coliform/100 ml) = (coliform colonies counted ×100)/volume of sample filtered (ml)

Water samples were categorized into four risk categories/classes based on the coliform count: Conformity <1 cfu/100 ml, Low 1–10 cfu/100 ml, Intermediate 11–100 cfu/100 ml and High >100 cfu/100 ml, as per the WHO guideline (24).

### QA/QC (Quality Assurance/Quality Control)

After water sample collection, all the samples were placed in ice immediately and transported to the laboratory in ice-cooled condition at 4°C. After arrival to laboratory, all the samples were refrigerated at 4°C and analyzed within 10 h of collection. Previous research showed that holding the temperature below 10°C for 12 h has little effect on the *E. coli* concentration (21). For quality assurance of chemical parameters analysis through ICP-MS, a standard solution was used, provided by Perkin-Elmer NexIon 300X, USA. However, for microbiological quality control and assurance WHO standard guidelines from 2008 and APHA 1999 (Standard method for examination of water and waste water) were followed to ensure the reliability of laboratory results (23, 25).

### Statistical Analysis

Results were analyzed using Microsoft Excel (Office 365). Wilcoxon signed-rank test was performed to compare the log concentration of *E. coli*, total coliform and *Enterococci* between spring water, community reservoir and household water samples. One-way ANOVA was used to compare colony forming unit (cfu) in different water sources and significance of the difference in the physicochemical parameters. The piper plot was used to categorize the water based on total hardness. The piper plot was constructed using AquaChem v 5.0. Pearson correlation was used to determine the correlation between the different physicochemical parameters of East, South, North and West districts' water samples. The correlation coefficient (*r*) was calculated using Microsoft Excel (office 365) and the correlation plots were prepared using R studio (package: corrplot and PerformanceAnalytics) (26). Multivariate analysis like principle component analysis and hierarchical clustering are useful tools for analyzing the water quality parameter and establishing correlation between physicochemical and microbiological parameters (12, 27). Principle component analysis and hierarchical clustering (based on UPGMA method) were done using *R* statistics to categorize the water source of four districts based on physicochemical characteristics and coliform count.

## Results

### Microbiological Water Quality

#### Spring Water

**Figure 4:**
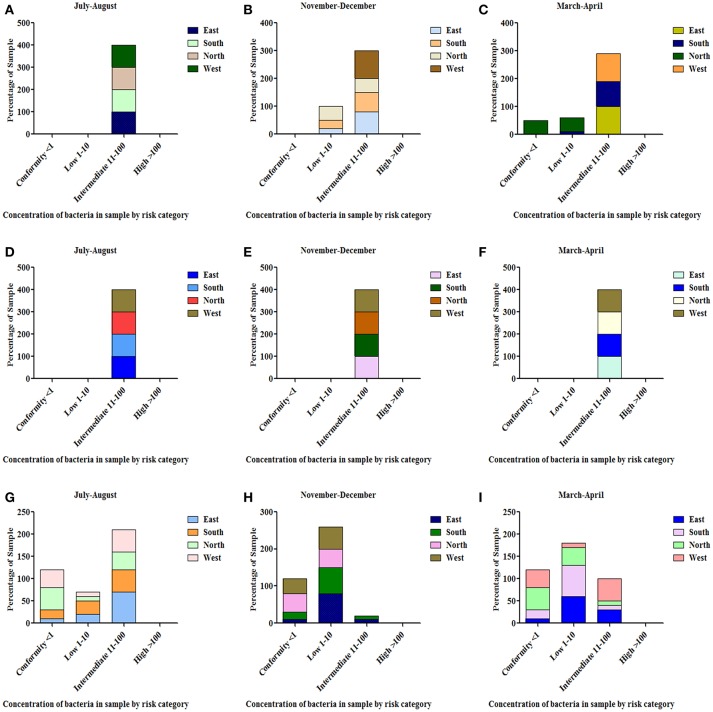
Characterization of water samples by risk category based on total coliform concentration. (<1 = conformity; 1–10 = low; 11–100 = intermediate; > 100 = high risk level for potable use). Total coliform concentration was determined in the samples of spring water **(A–C)**, community reservoir **(D–F)**, and household water **(G–I)** during different seasons. Note the total samples of each district (100 percent) are distributed in different risk categories. The microbial contamination of water sources varied in different seasons and generally peaked in the rainy season during July-August. The order of water contamination level in different district was East > South > West > North.

**Figure 5:**
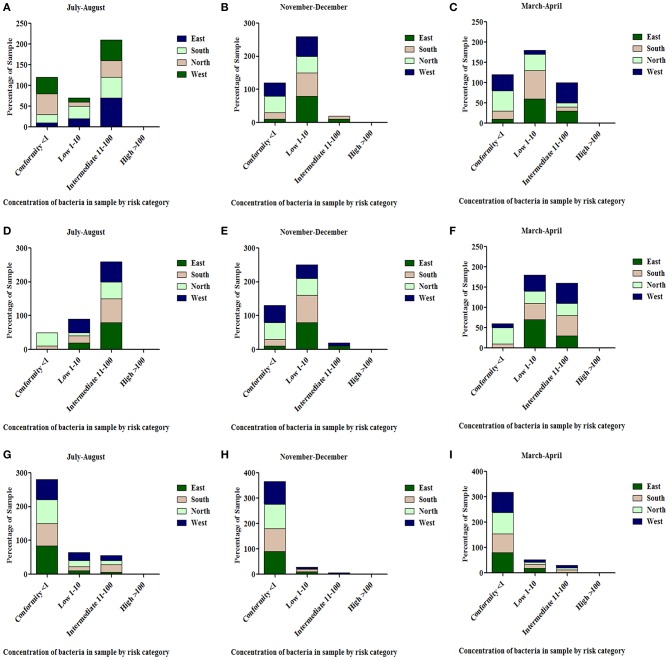
Characterization of water samples by risk category based on *E. coli* concentration. The *E. coli* concentration was determined in the samples of spring water **(A–C)**, community reservoir **(D–F)**, and household water **(G–I)** in different seasons. Note the total samples of each district (100 percent) are distributed in different risk categories. The *E. coli* levels showed seasonal variation. It generally peaked in all types of sources during the rainy season (July-August), while at other times it showed minor variations.

All the spring water (100%) in the month of July–August (rainy season) were in the category of intermediate risk level as per the classification of the WHO (21, 24); 80% of the spring water from East Sikkim in the month of November–December was found to be at an intermediate risk level and 20% were at a low risk level, while 70% of the Spring water from South Sikkim in November–December was at an intermediate risk level and 30% was at a low risk level. Meanwhile, 50% of spring water from North Sikkim showed an intermediate risk level and 50% showed a low risk level for total coliform concentration in the entire season (Figure 4). Water samples from West Sikkim came under the intermediate risk level in the all three seasons. It was also found that 90% of spring water from East Sikkim was contaminated with *E. coli*, followed by South Sikkim (80%) and West Sikkim (60%), and North Sikkim showed the least contamination (50%) (Figure 5). The results showed spring water samples were highly contaminated during the month of July–August in all four districts while they remained least contaminated in the month of March–April.

#### Community Reservoir Tank

In the case of community reservoirs, the rainy season (July–August) was the most contaminated season. It was found that all the community reservoirs were at an intermediate risk level with total coliform in these months (Figure 4), whereas in case of *E. coli*, East Sikkim showed the highest contamination, with 80% of reservoir water under an intermediate risk level. North Sikkim showed the least contamination, with 50% of reservoir water at an intermediate risk level (Figure 5). Winter (November–December) was the least contaminated season, where majority of the reservoirs (50–80%) were at a low risk level. Only 0–10% reservoirs were found in the intermediate risk level among the four districts during the winter season (Figures 4, 5).

#### Household Water

Household water samples were also contaminated with coliform bacteria above the permissible limit of WHO standard guidelines. The rainy season contributed the most contamination to the water. East Sikkim was highly contaminated, with 60% of samples belonging to the intermediate risk level, while North Sikkim showed the least at 34%, in the case of total coliform (Figure 4). In the case of *E. coli*, South Sikkim was found to be the most contaminated with 22% samples under intermediate risk level, and East Sikkim showed the lowest with 6% of samples at an intermediate risk level (Figure 5). However, as compared to springs and community reservoirs, most of the household water samples conformed with the low risk level category (80–90%).

### Household Water Treatment Profile

**Figure 6:**
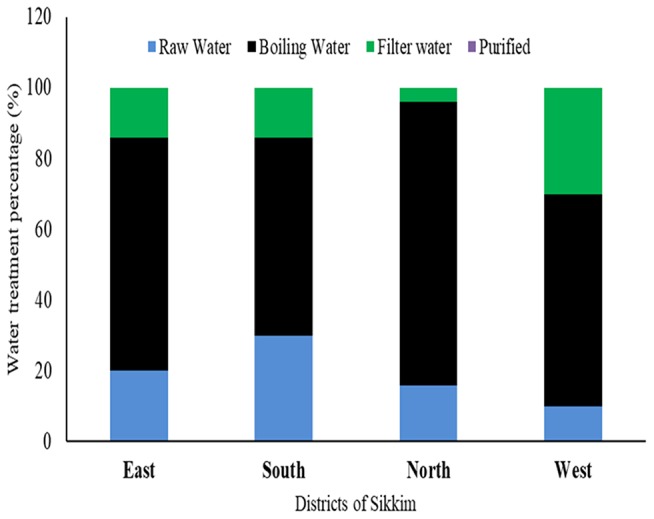
Different water treatment profiles among the households of different districts of Sikkim. Boiling is the most common method used by residents prior to consumption.

Most of the study subject households (10 villages ×4 districts ×5 households from each district = 200 households) had never used any kind of standardized treatment or filtration process prior to using water for any purpose. Boiling was found to be the only or the major form of decontamination used by the households in all four districts of the state (East: 66%; West: 60%; North: 80%; South: 56%), only a small percentage of the people used filtration prior to its consumption or use for any other purpose (East: 14%; West: 30%; South: 14%, and North: 4%). A significant proportion of the population consumed the raw water directly from the source (spring water) (East: 20%; West: 10%, South: 30%, and North: 16%) (Figure 6).

**Figure 7:**
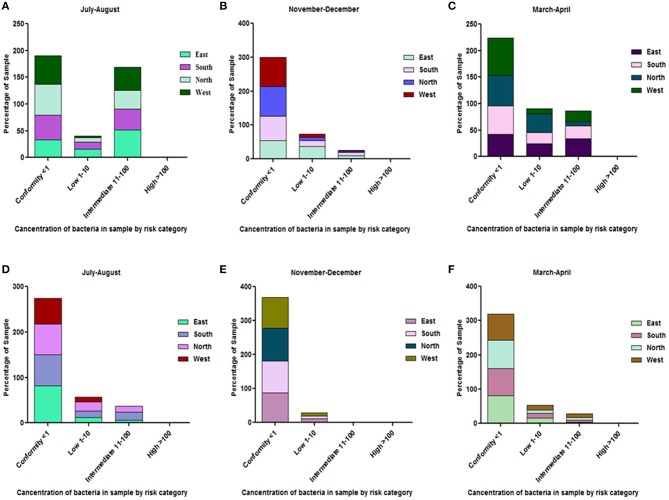
Risk category characterization of household water samples which used boiling as a method of water sanitation. **(A–C)** depict total coliform concentration while **(D–F)** depict *E. coli* concentrations in the household water. The total samples of each district (100 percent) are distributed in different risk categories. Most of the household water was of conformity level. However, the contamination level generally increased during the months of July-August (rainy season).

Surprisingly, the stored household water which had been boiled previously recorded total coliform and *E. coli* counts above the permissible limit. However, total coliform and *E. coli* counts in all the household samples which were boiled prior to consumption were found to be in the range of the intermediate to low risk category. The rainy season, or July–August, showed the highest contamination. East and South Sikkim showed the highest levels of contamination compared to the West and North (Figure 7).

**Figure 8:**
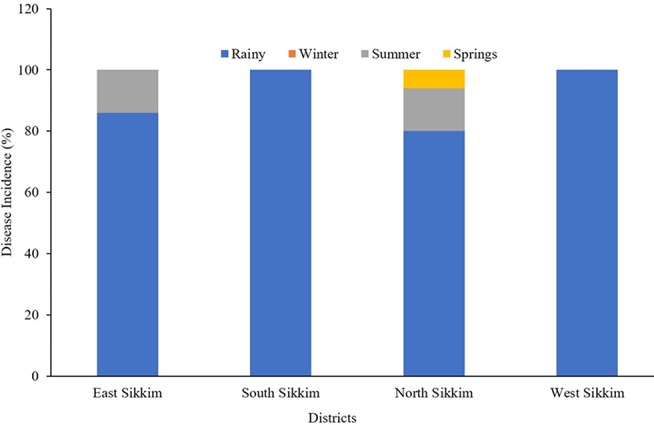
The occurrence (percentage) of disease based on community response. The most disease-prone time in Sikkim was rainy season.

From the survey conducted, it can be summarized that the rainy season was responsible for most of the water-borne diseases. Based upon the response of the surveyed population, it was found that 86% of overall water-borne disease incidents recorded from the East Sikkim had occurred in the rainy season compared to about 14% for the summer. In the case of South and West Sikkim, the rainy season recorded 100% of the reported water-borne diseases. North Sikkim incidentally recorded 80% of the disease occurrences in the rainy season while 3% of disease occurrences were recorded in the spring season (Figure 8).

### Physicochemical Parameter

The concentration of TDS in the four districts varied from 13.5 to 41.5 mg/l, with East Sikkim having the highest TDS of 41.5 mg/l and the North having the lowest (13.5 mg/l). The total hardness (TH) ranged from 21 to 37 mg of calcium carbonate equivalent per liter. East Sikkim had the highest TH (37 mg/l) while lowest was measured in North Sikkim (21 mg/l). All the water samples were neutral in nature with a pH range of 6.52–7.18. The temperature of the water ranged between 18.6 and 20.8°C. Turbidity recorded was in the range of 1.5–2.6 NTU. There was no statistically significant difference between the physical parameters like pH, temperature, TDS and TH at *p* < 0.05 except electric conductivity. Electric conductivity (EC) was recorded in the range of 96.0–188.1 μS/cm. The highest electric conductivity (188.1 μS/cm) was recorded from South Sikkim and the lowest (96 μS/cm) was recorded from North Sikkim (Table 1).

**Table 1:** Comparative physicochemical parameter with the standard guidelines of World Health Organization (1), BIS (28), and CPCB (29).

**Parameters**	**WHO**	**BIS**	**CPCB**	**E**	**S**	**N**	**W**
pH	6.5–8.5	6.5–8.5	6.5–8.5	7.18	6.52	7.01	7.1
Turbidity NTU	NA	10	10	2.604	2.124	1.819	1.503
Conductivity (mg/l)	NA	NA	2,000	184.3	188.1	96	177.2
Alkalinity (mg/l)	NA	NA	600	26	25	24	23
Total hardness (mg/l)	500	500	600	25	35	21	37
Iron (mg/l)	0.1	0.1	1	10.869	1.615	10.823	4.763
Chlorides (mg/l)	200	200	1,000	16	24	13	18
Calcium (mg/l)	75	75	200	35.205	8.525	25.71	13.415
Magnesium (mg/l)	50	30	100	120.896	23.532	58.829	43.323
Copper (mg/l)	1	0.05	1.5	3.052	3.206	1.941	2.106
Fluoride (mg/l)	1.5	0.6–1.2	1.5	0.038	0.038	0.036	0.036
Mercury (mg/l)	0.001	0.001	No relaxation	0.007	0.00375	0.004	0.001
Cadmium (mg/l)	0.005	0.01	No relaxation	0.026	0.011	0.012	0.024
Selenium (mg/l)	0.01	0.01	No relaxation	0.644	0.578	0.686	0.526
Arsenic (mg/l)	0.05	0.05	No relaxation	0	0	0	0
Lead (mg/l)	0.05	0.05	No relaxation	0.04	0.023	0.036	0.051
Zinc (mg/l)	5	5	15	0.208	0.76	1.943	0.403
Chromium (mg/l)	NA	NA	No relaxation	1.517	0.249	1.16	1.069
Nitrate (mg/l)	50	–	–	9.4	8.8	9.4	9

**Figure 9:**
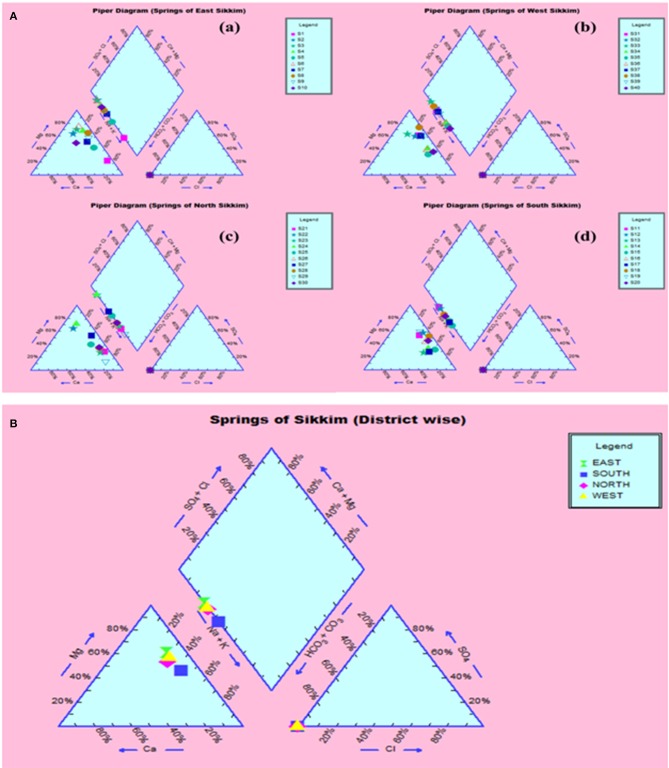
Piper analysis showed water from all four districts was Ca-Mg-HCO^3−^ type. **(A)** District-wise classification (a) East, (b) West, (c) North, (d) South. **(B)** Collective classification.

The compositional distribution of chemical constituents using Piper analysis (30) showed that water from four districts was dominated with cation Mg^+^. There was no significant variation in chemical constituents as a function of sample location. The anionic composition was dominated by bicarbonate (HC${\text{O}}_{3}^{-}$) ions. Based on the Piper analysis, water can be classified as Mg-HC${\text{O}}_{3}^{-}$ and categorized as shallow fresh groundwater (Figure 9). The water chemistry analysis also showed richness in Mg and Fe. Concentrations of Mg and Fe ranged from 43.320 to 120.896 mg/l and 1.615 to 10.869 mg/l, respectively. Copper was present in the concentration range of 1.94–3.05 mg/l. Surprisingly, water samples were recorded with traces of mercury, selenium and chromium in the ranges of 0.001–0.007, 0.526–0.644, and 0.240–1.517 mg/l, respectively (Table 1).

**Figure 10:**
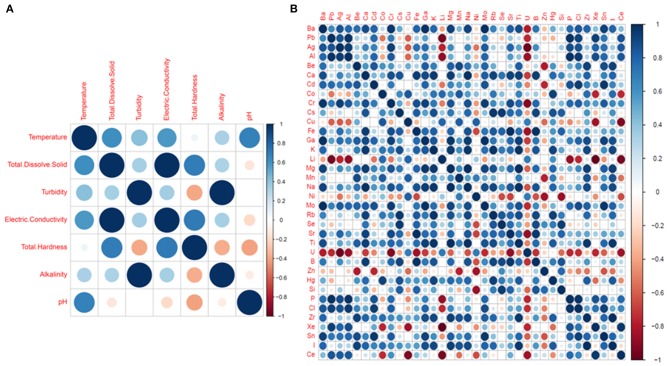
Pearson correlation plot between physicochemical parameters **(A)** Basic parameters and **(B)** Chemical elements. The dark blue colors define high positive correlation and dark red color defines high negative correlation.

**Figure 11:**
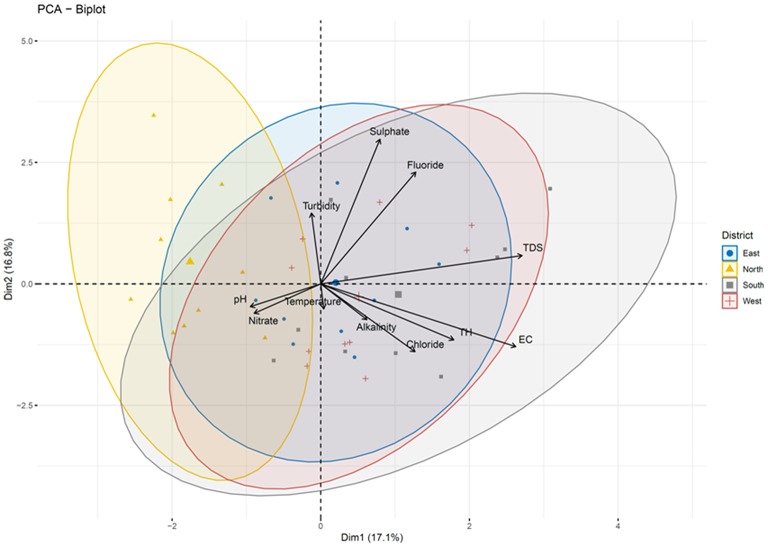
Biplot analysis of the physicochemical parameters across the four districts. Clustering based on principle component analysis showed distinct clusters for four districts defining the differences in physicochemical parameters of water samples.

Pairwise Pearson correlation analysis between different water quality parameters of the four districts showed interesting correlations. The correlation coefficient (r) was determined between temperature, TDS, turbidity, electric conductivity, total hardness, alkalinity and pH (Figure 10). The correlation matrix showed a positive correlation between EC and TDS (*r* = 0.998/1.000, *p* < 0.05) and a negative correlation between pH and TDS (*r* = −0.139/1.000) (Supplementary Table S4; Figure 8). A strong correlation was also observed between alkalinity and turbidity (*r* = 0.993/1.00, *p* < 0.05), while pH, TH and alkalinity were found to be negatively correlated. The correlation coefficient (*r*) between alkalinity and pH was *r* = −0.108/1.000. Similarly, total hardness was negatively correlated with alkalinity (*r* = −0.367/1.000) and pH (*r* = −0.405/1.000) (Supplementary Table S4). Correlation analysis between the chemical parameters showed that the concentration of Pb and Al covaried, with a correlation coefficient r = 0.975 /1.000, *p* < 0.05. A high positive correlation was also found between the concentration of silver and aluminum (*r* = 0.948/1.000), gallium and barium (*r* = 0.988/1.00, *p* < 0.05), sodium and calcium (*r* = 0.980/1.000, *p* < 0.05), chromium and barium (*r* = 0.996/1.00, *p* < 0.05), and magnesium and potassium (*r* = 0.96/1.000, *p* < 0.05). A strong correlation was also observed between potassium and calcium (*r* = 0.95/1.0, *p* < 0.05) as well as between magnesium and sodium (*r* = 0.99/1, *p* < 0.05). A strong negative correlation was found between Li, U, Xe, Al, and Ag [*r* (Li and Xe) = −0.99/1.000; (*p* < 0.05), *r* (Li and Ag) = −0.769/1.000]; *r* (Li and Al) = −0.880/1.000 (*p* < 0.05)], U and Ag [*r* = −0.97/1 (*p* < 0.05)] (Supplementary Table S4; Figure 10). Principle component analysis was done using the biplot method to group the water based on physicochemical characteristics and total coliform count. Clustering based on PCA-biplot analysis showed a correlative cluster among the spring water sources of East, West and South Sikkim. North Sikkim formed a different cluster, as expected from the difference in physicochemical and total coliform count (Figure 11). Principle component 1 (PC1) or Dimension 1 (Dim 1) explains the variance of PC2 (17.1%) and Dim2 (16.8%) among the variables. The correlation based on the loadings showed positive correlation between pH, nitrate, alkalinity, chloride, total hardness, and electro conductivity (Figure 11).

**Figure 12:**
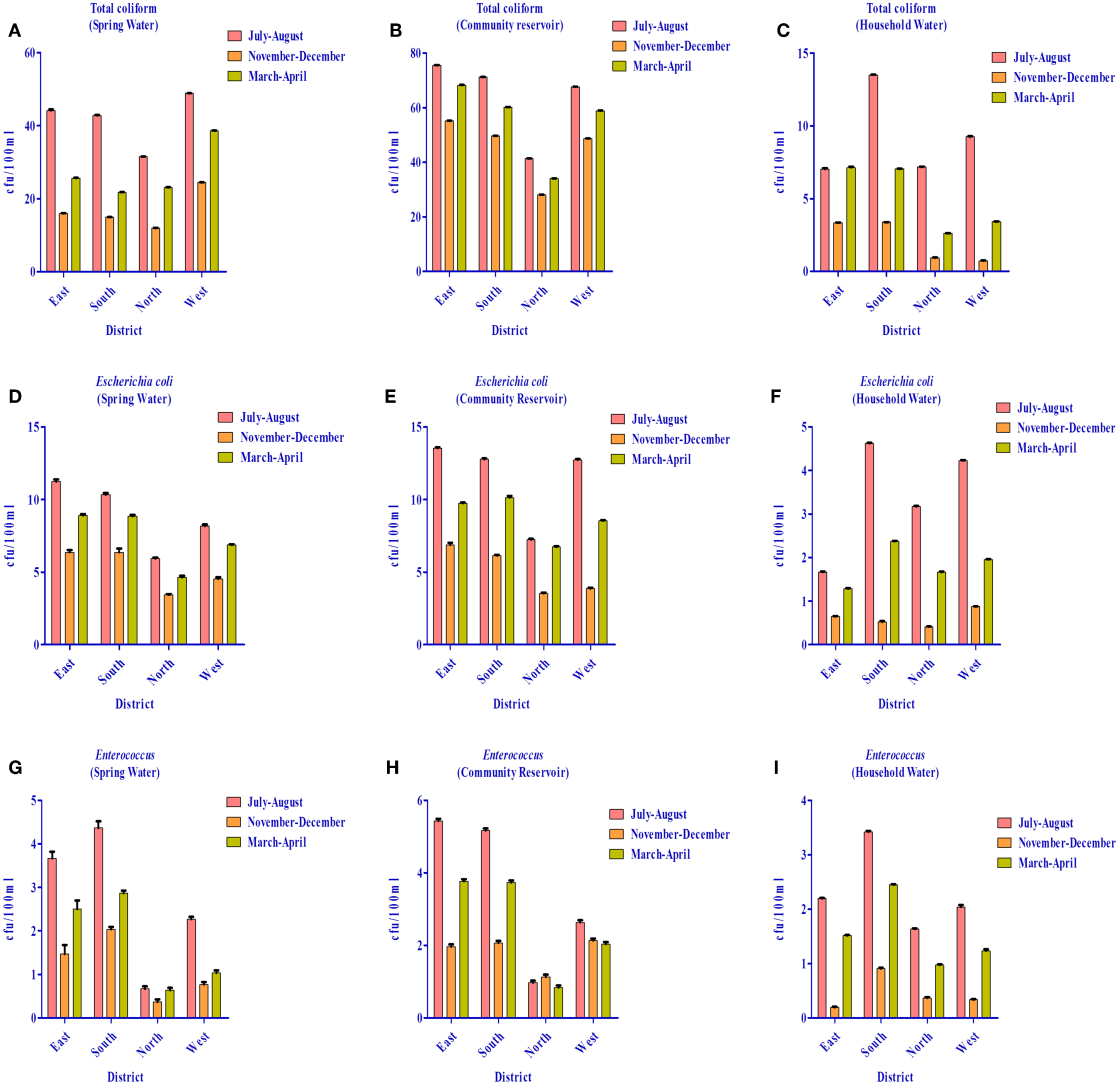
Distribution of total coliform (TC), *Escherichia coli* (EC) and *Enterococcus* (EN) bacteria in the springs, community reservoir tank, and household water in the four districts during different seasons. **(A)** TC in springs **(B)** TC in community reservoir **(C)** TC in household water **(D)** EC in springs **(E)** EC in community reservoir **(F)** EC in household water **(G)** EN in springs **(H)** EN in community reservoir and **(I)** EN in household water. It was demonstrated that the community reservoir becomes more contaminated than spring water, and household water was the least contaminated. July-August showed more contamination as compared to other seasons.

As per WHO guidelines (1) for potable water, indicator organisms must not be present (= 0 cfu/100 ml) to qualify for potable use. However, all three indicator bacteria assessed were detected in the water distribution system of different districts (i.e., spring water, community reservoir, and household water) (Figure 12). Spring water from East Sikkim was found to have total coliform count in the range of 16–44.4 cfu/100 ml. The total coliform count in South, North and West Sikkim ranged from 14.9–42.8 cfu/10 0 ml, 11.9–31.5 cfu/100 ml and 24.4–48.9 cfu/100 ml, respectively. The rainy season (July–August) accounted for the highest total coliform count (cfu/100 ml) in all the districts, i.e., East = 44.4 cfu/100 ml, South = 42.8 cfu/100 ml, North = 31.5 cfu/100 ml, and West = 48.9 cfu/100 ml. Community reservoirs were recorded with an astoundingly high number of total coliform bacteria, with a highest average mean of 75.6 cfu/100 ml in East Sikkim in the rainy season followed by South (71.1 cfu/100 ml), West (67.6 cfu/100 ml), and North Sikkim (41.4 cfu/100 ml) (Supplementary Table S5). Household water was recorded with the least number of coliform when compared with spring and community reservoirs. The mean total coliform concentrations for household water for the different districts during the rainy season were as follows (cfu/100 ml): East = 13.36 cfu/100 ml, South = 13.52 cfu/100 ml, West = 9.28 cfu/100 ml, and North = 7.20 cfu/100 ml. The lowest number of coliforms was detected in the winter season among the three water sources.

The highest *E. coli* count was recorded from the community reservoirs of East Sikkim (13.5 cfu/100 ml) in the rainy season and lowest was recorded from the household water from North Sikkim (0.4 cfu/100 ml) in winter season. *Enterococcus* was also recorded in high number from community reservoirs in the rainy season. District-wise distribution of *Enterococcus* detection showed the highest count from East Sikkim (5.5 cfu/100 ml) followed by South (5.2 cfu/100 ml), West (2.6 cfu/100 ml), and North Sikkim (1 cfu/100 ml), respectively (Figure 12). Cluster analysis based on the mean concentration of total coliform, *E. coli* and Enterococcus formed three distinct sub-clusters. South and West Sikkim formed one single cluster, defining the similarity in the concentration of indicator bacteria, while East and North Sikkim formed two separated clusters, being the two outliers, East with highest contamination and North with lowest (Supplementary Figure S1).

## Discussion

Water is essential to life, thus access to microbiologically and chemically safe water is important to human health. Providing safe and clean water is a basic norm in developed countries but many people, especially in developing countries, still struggle to get access to safe water (31, 32). Water systems with inadequate quality control and sanitation in place act as a vehicle for the dissemination of different pathogenic microorganisms originating from the feces of humans or wildlife. Although huge efforts are made to provide safe drinking water, there are still 1.1 billion people worldwide who are consuming water contaminated with fecal microorganisms (14). Detecting each waterborne pathogen is complex; therefore, a standard reproducible microbial water quality test was developed by the International Organization for Standardization (ISO) to test the water quality. In this method, detection of certain groups of bacteria acts as an indicator for fecal contamination (1). The most widely used indicators are total coliform, *E. coli* and *Enterococci* (33). However, not only the bacterial pathogens but several physicochemical parameters also determine the water quality of a region. There are standard guidelines by both WHO and BIS (Bureau of Indian Standards) for the safe and hygienic water index (1, 28).

Sikkim is an Eastern Himalayan state with high altitudes as topological features. The majority of the population depends on the naturally occurring spring waters for their day to day use. To maintain the continued supply of spring water, the government of the state made community reservoirs which are responsible for storing the spring water and supplying it to the households of an area. Recent outbreaks of several waterborne diseases in different parts of the state have raised concern regarding the quality of the water consumed by its residents. As per a recent report released by the Ministry of Health and Family Welfare, Government of India, 2017, several major waterborne diseases, e.g., acute diarrheal diseases, enteric or typhoid fever, cholera, etc., had been consistently reported from Sikkim. A total of 39,983; 53,295; and 46,289 cases of acute diarrheal diseases were reported during the years 2014, 2015, and 2016, respectively. With regard to enteric fever (typhoid) occurrences, a total of 716, 453 and 474 cases were reported from Sikkim in years 2014, 2015 and 2016, respectively [(34), http://www.indiaenvironmentportal.org.in/files/file/NHP_2017-1.pdf] (Supplementary Data Sheet 1). In this study, we have tried to evaluate the water quality of four districts of the state with respect to both physicochemical and microbiological profile. A structured questionnaire was used to gather information on different filtration techniques used by the households along with recent diseases. We found that filtration and purification processes are one of the less common practices among the hill population of the state. Boiling water is the only common practice used for purifying water before consumption. On average 80% of the population of North and 66% of the East Sikkim uses boiled water for drinking. Similarly, 56% of South and 60% of West Sikkim use boiled water for drinking. There are reports suggesting that boiling can provide effective protection against *Vibrio cholera* infections, *Blastocystis*, protozoal infections, viral infections, and non-specific diarrhea (35). Our observations were in close agreement with previous findings where household water subjected to boiling prior to drinking was recorded with the least number of microbial contaminants (Figure 7). Only 10% of household water samples were found to be safe for drinking, where direct spring water/community reservoir water was used as such (coliform count <1 cfu/100 ml), whereas 33.3% water samples fell in the safe category wherever boiling was applied before using it for the drinking purposes (Supplementary Table S5). The *E. coli* count was also found to be significantly low in boiled water. A total of 81.81% of household water samples in East Sikkim showed *E. coli* counts <1 cfu/100 ml (Supplementary Table S5). In research by Clasen et al. (36), it was shown that boiling was associated with a 97% reduction in the geometric mean of thermotolerant coliforms (TTC) (*p* < 0.001). The study indicated that despite knowing the benefits of water boiling, a significantly large proportion of population was still using spring water as a direct source without any treatment. A total of 20, 10, 30, and 16% of the population from East, West, South, and North Sikkim were still using spring water as a direct source of drinking. This indicates the need for awareness and outreach programs to educate the people about different water treatment methods and their importance.

The community reservoirs which were developed to work as storage and supply stations for the households in village level blocks were the most contaminated ones. The high numbers of fecal coliforms in samples of community reservoirs indicate severe human or fecal contamination at the spring or during its transportation to reservoirs and households. The correlation between the total coliform count of spring water, community reservoir and household water showed a significant relationship between their contamination sources. Community reservoirs showed a high positive correlation with spring water (*r* = 0.90; *p* < 0.05). It indicates the contamination at spring water sources can affect the contamination level in community reservoirs as there was no treatment between transport (Supplementary Figure 2). The high number of coliform bacteria in the water source would be a concern for the state, as increased coliform counts in the water source can indicate presence and/or proliferation of other opportunistic waterborne pathogens such as *Klebsiella, Enterobacter*, and *Pseudomonas* (37). Coliform generally forms biofilm at the distribution chanels (pipes) and provides an anchoring and proliferation site for opportunistic pathogens (37–39).

Based on the presence of coliform bacteria, water sources of East Sikkim were found to be the most contaminated while those of the North Sikkim were the least contaminated (Figure 11). There was no significant statistical difference among the water sources of the four districts (*p* > 0.05) with respect to total coliform count. This means that all the four districts were in a similar alarming state. The UPGMA cluster analysis using total coliform count showed three distinct clusters. South and West Sikkim formed a single cluster owing to the similarities in concentration of coliform count. North and East Sikkim were outgroups. North Sikkim had the lowest and East Sikkim had the highest coliform count (Supplementary Figure S1). The severity of water contaminants in East Sikkim is of greater concern as it is the main economical, industrial and educational hub for the state and has the highest population (45.3%) (40). Inversely, it also indicates that severe contamination is happening due to the increased animals or human activity, which needs to be taken care urgently. North Sikkim has the lowest population in comparison to other districts, which may be the reason for less human contact and less human-made contamination. But this report suggests a serious immediate health risk which could happen until and unless government and public healthcare personnel formulate policy structures to protect the important potable water sources of the state from human and animal interventions.

The physicochemical analysis showed that the concentration of most of the elements was within the standard permissible limits as per the WHO guidelines for potable water. The presence of a few toxic metalloids like cadmium, slenium, mercury and chromium were found above the permissible standards of the WHO, BIS, and Central Pollution Control Board (CPCB) (Table 1). Detection of these heavy metals poses a serious concern over the health of Sikkimese people. The International Agency for Research on Cancer (IARC) classified the elements As and Cd as carcinogens. Cd is related to cancer skin damage, kidney damage and heart disease. Geological deposition of Cd serves as its source in ground and surface water. High concentration of Cd in water sources affects the lungs and kidneys (41), disturbs the bone metabolism and the endocrine system, and deforms the reproductive tract (42). Consumption of Cr-contaminated water can cause stomach cancer and anemia (43). Se causes high blood cholesterol. Lead (Pb) is related to anemia. It is a cumulative toxicant that affects multiple metabolic pathways and is particularly harmful to young children. Consumption of high concentrations of lead for long periods of time can affect the normal functioning of the brain, kidneys, liver and bones (44). There are several studies that indicate a high concentration of lead can be a reason for stunted growth in children (45, 46). Mercury (Hg) causes kidney and liver damage. It is a very toxic element that can be found in the form of elemental mercury, inorganic mercury and organic mercury like methyl mercury. In minor quantity methyl mercury affect the cardiovascular system and can cause vomiting, blisters in upper gastrointestinal tract, constipation, abdominal pain and gastritis. Blood-absorbed mercury can pass to the placenta and affect the nervous system of developing fetuses. Continuous exposure to Hg leads to its accumulation in thyroid and subsequent infection (42). Cu is reported to be associated with gastrointestinal disease (47). As parts of different districts indicate the presence of different toxic metals in drinking water, policy makers may devise ways to mitigate the effect of the presence of these metals in the drinking water on the health of the local population. Local populations may be suggested to start using suitable purification systems. Additionally, a survey of the area may be conducted to correlate the presence of these elements in the drinking water with the incidences of the diseases associated with the intake of toxic metals.

The water sources' excessive physical (turbidity, electric conductivity and alkalinity), chemical (iron, copper, mercury, cadmium, and magnesium) and biological parameters (total coliform*, E. coli* and *Enterococcus*) can negatively impact human health (Tables S2, S4, S5). As discussed in the results section, some of the chemical parameters like concentration of iron, magnesium, copper, mercury, cadmium, selenium, and lead were above the WHO permissible limits. The higher concentration of these chemicals and deviation from permissible limits of physicochemical and biological parameters are a health hazard. Along with the physicochemical parameters, the biological parameters like total coliform, *E. coli* and *Enterococcus* were above the WHO limits in water sources (Figure 12) (1). The presence of coliform bacteria in high concentrations in the water samples of both springs as well as community reservoirs indicated that water was contaminated with warm-blooded animals' fecal matter, such as humans or wild animals, and not safe for drinking. This makes urgent the need for proper protection of the major water sources at the supply level or at the source of origination to minimize the human and animal contact. Household water samples were also contaminated with fecal coliform bacteria above the permissible standard, which indicates the severity of the situation and the necessity of the dissemination of awareness about different sterilization and filtration processes at the household level. The seasonal study showed that the rainy season was a major contributory factor in microbial contamination and needs much more attention. Elevated pollution and microbial contamination in the rainy season indicate the mixing of runoff water with animal fecal materials with water sources at some level. This calls for undertaking immediate steps to protect potable water sources from runoff water during heavy rainfall. High contamination in East Sikkim in the rainy season suggests the possibility of mixing of the runoff water with the spring water sources. Increased rate of road, building and pavement construction in East Sikkim, as capital of the state, has reduced the land surface available for soaking up the runoff water, leading to increased flooding and its mixing with spring water. As an immediate precautionary measure, especially during the rainy season, proper chlorination and sterilization of drinking water before supplying to the households is suggested. Based on the water quality, the following water management options are recommended at different levels of the drinking water supply chain in the state of Sikkim:

- Proper physicochemical and biological testing of the springs before selecting it as a source of supply.
- Putting fences around the springs to prevent access to the source from animals.
- Periodical inspection of the community reservoirs for cracked well casting, broken or missing wells, and corrosion of the connecting pipes.
- Installations of sanitary caps/lids to prevent unauthorized use and access to the reservoir tank.
- Disinfection of the reservoirs quarterly with bleach and hypochlorite as per standard protocols.
- Periodical inspection of the reservoir water for the indicator bacteria to assess the water quality.
- Slope the area around the reservoir wells to drain surface runoff away from the reservoir tank.
- Keep proper records of the well maintenance services, including chlorination, disinfection, and microbial counts to devise a course of action.
- Prevent disposal of any kind of waste material in or near the springs/reservoir tank.
- At a household level, proper boiling of water before consumption should be promoted.
- Public awareness programmes promoting use of simple sand gravel filters with alum (as they are easily available raw materials) should be implemented by Government agencies/NGOs. Sand gravel filters can filter out microbes such as bacteria and protozoa, whereas aluminum would cause coagulation of dart which can be easily removed by decantation.
- Slow sand gravel filtration and sand gravel coal filtration could be used for the removal of trace heavy metals. Egg shell powder with chitosan can also be employed for the removal/ filtration of trace heavy metals like lead and copper.

Our study points to possible fecal or surface runoff contamination of the drinking water sources in all districts of the state as indicated by microbiological indicators like total coliform and *E. coli* counts. The evaluated microbiological indicators were found to be well above the permissible limits of WHO for potable water. Though bacteria are naturally present in the environment like water bodies, soil, ponds and lakes, the level of coliform bacteria is generally considered as an indicator of the level of fecal contamination in water bodies and the associated health risk (48). The presence of fecal coliforms above a permissible level is known to cause ailments like nausea, cramps, diarrhea, vomiting, and headache. In rare occasions, high microbial contamination of the drinking water is associated with hemolytic uremic syndrome, which is a serious kidney disease and may cause lifelong complications (48). The detection of a high number of coliform bacteria in the water distribution network of Sikkim suggests fecal contamination of water, so other pathogenic bacteria like *Vibrio, Salmonella, Shigella*, and parasites like *Cryptosporidium, Giardia* may be also present. *V. cholera, Shigella*, and *Salmonella* cause diseases like cholera, bacillary dysentery and typhoid fever, respectively (49). Incidentally, several cases of acute diarrheal and enteric fever (typhoid) had been reported from Sikkim in the recent years, such as during 2014–2016 (34). It prompts us to suggest possible contamination of the water distribution network of the state with the fecal material that needs to be immediately checked to improve the health index of the state residents.

## Conclusion

In Sikkim, the community considers springs as a holy pristine source of water which is safe for drinking and other household purposes. However, recent outbreaks of several waterborne diseases like diarrhea and typhoid have prompted us to analyze the water quality of springs. Major findings can be summarized as follows:

- Most of the spring and community reservoir water came under the intermediate health risk level category as per guidelines for drinking water quality (1).
- The physicochemical parameter analysis revealed that though most of the water samples had basic elements within the safe limits, the traces of heavy metals were well above the WHO, BIS, and CPCB guidelines, 2017. *Heavy metal contamination like lead, chromium and mercury indicates an existing health hazard*.
- The microbial parameters in the water samples of the springs and the community reservoirs were also higher than mandated by WHO guidelines for drinking water. The community reservoir water samples were found to be more contaminated than the spring water. *High contamination of coliforms indicates possible mixing of fecal materials with the source water*.
- Interestingly, some of the household water samples that employed boiling as a water sanitization method were found to be safe for drinking as per the microbiological quality, i.e., coliform counts guidelines set by WHO. *This indicates that boiling could serve as a good primary water sanitizing method for microbial contaminants*.
- The springs of East and South Sikkim were more contaminated than the other two districts. The lowest microbial load was found in the North Sikkim.
- Water sources are not safe for drinking and the situation's compounding factors need immediate attention. *At the state level, regular checking of physicochemical and biological parameters of the water source, its maintenance to prevent possible human and animal contact along with treatment and purification of water as per the need before its distribution should be ensured to avoid huge health cost*.
- General chlorination and disinfection of spring water and community reservoir should be practiced before its supply to households. *The community needs to be educated about general hygiene, different methods available for household-level water treatment and the importance of clean water for children and their health to avoid impending health disaster associated with the unsafe water supply*.

## Author Contributions

HT designed and supervised the study. AS performed the experiment. AS, SD, and SS analyzed the data and prepared the manuscript. VG and NP helped in the sample collection, survey, and lab experiments. SK helped in manuscript preparation. YL helped in the selection of study area and official documentation regarding the study.

### Conflict of Interest Statement

The authors declare that the research was conducted in the absence of any commercial or financial relationships that could be construed as a potential conflict of interest.

## Supplementary Material

Supplementary Table S1 Name of sample collection site with latitude and longitude.

Supplementary Table S2 Spring details.

Supplementary Table S3 Structured questionnaire.

Supplementary Table S4 Principle Component analysis of statistical data of physicochemical parameters.

Supplementary Table S5 Microbiological analysis of water samples.

Supplementary Figure S1 Cluster analysis based on the mean concentration of total coliform, *E. coli* and *Enterococcus*.

Supplementary Figure S2 The correlation between the total coliform count of spring water, community reservoir and household water.

Supplementary Data Sheet 1 Report of several major waterborne diseases released by Ministry of Health and Family Welfare, Government of India.
