# Supplementary material for: Physicochemical Parameters and Alarming Coliform Count of the Potable Water of Eastern Himalayan State Sikkim: An Indication of Severe Fecal Contamination and Immediate Health Risk
Source: Front Public Health. 2019 Jul 10;7:174. doi: 10.3389/fpubh.2019.00174 (PMC6636254; doi:10.3389/fpubh.2019.00174)
Supplement: Supplementary Table S2 — Spring details. [file Table_2.DOCX]

| S. No. | District | Spring | Latitude | Longitude | Altitude (m) | No of population dependent |
| --- | --- | --- | --- | --- | --- | --- |
| 1 | East | Bhotey Khola | 27.41 | 88.62 | 1411 | 3000 |
| 2 | East | Kali Khola | 27.21 | 88.59 | 1036 | 2000 |
| 3 | East | Gurung Khola | 27.20 | 88.59 | 1047 | 620 |
| 4 | East | Devithan Dhara | 27.21 | 88.59 | 1136 | 350 |
| 5 | East | Pam Dhara | 27.20 | 88.66 | 832 | 500 |
| 6 | East | Pangu Dhara | 27.19 | 88.33 | 1207 | 300 |
| 7 | East | Navey | 27.27 | 88.48 | 1602 | 360 |
| 8 | East | Bhotay Khola | 27.26 | 88.58 | 1109 | 320 |
| 9 | East | Gairay Dhara | 27.38 | 88.50 | 1513 | 130 |
| 10 | East | Hel Khola Main | 27.20 | 88.57 | 1768 | More than 5000 |
| 11 | South | Phalado Dhara | 27.15 | 88.31 | 1018 | 327 |
| 12 | South | Ratmatey Dhara | 27.12 | 88.34 | 1121 | 600 |
| 13 | South | Took Dhara | 27.13 | 88.36 | 1007 | 1578 |
| 14 | South | Kami Dhara | 27.10 | 88.18 | 1146 | 1350 |
| 15 | South | Kalijhar Kholsa | 27.15 | 88.35 | 1706 | 1725 |
| 16 | South | Jorkaspai Source | 27.17 | 88.21 | 1849 | 468 |
| 17 | South | Kubalakha | 27.26 | 88.30 | 937 | 500 |
| 18 | South | Devithaney Dhara | 27.14 | 88.26 | 955 | 1530 |
| 19 | South | Gurung Dhara | 27.22 | 88.26 | 994 | 350 |
| 20 | South | Shyam Pani | 27.08 | 88.20 | 1497 | 780 |
| 21 | North | Chungu Nonchay | 27.35 | 88.39 | 2738 | 450 |
| 22 | North | Samal Bong | 27.47 | 88.50 | 1375 | 852 |
| 23 | North | Sim Dhara | 27.30 | 88.33 | 1327 | 269 |
| 24 | North | Bakcha Dhara | 27.42 | 88.61 | 1359 | 100+ |
| 25 | North | Bitchu Chume | 27.66 | 88.71 | 2426 | 250 |
| 26 | North | Kadong Engram | 27.27 | 88.33 | 1019 | 210 |
| 27 | North | Namfong Keyung | 27.23 | 88.28 | 651 | 307 |
| 28 | North | Ravichyok-Nakyong | 27.51 | 88.50 | 965 | 527 |
| 29 | North | Devithan | 27.29 | 88.31 | 1325 | 205 |
| 30 | North | Kamtuk | 27.28 | 88.33 | 1121 | 190 |
| 31 | West | Jhordhara | 27.33 | 88.24 | 1488 | 495 |
| 32 | West | Bulbuley Pani | 27.15 | 88.47 | 1486 | 480 |
| 33 | West | Aaney Ghanti Khola | 27.11 | 88.11 | 12555 | 1500 |
| 34 | West | Dharapani Source | 27.07 | 88.11 | 759 | 175 |
| 35 | West | Ghurpisey Muhan | 27.10 | 88.14 | 1476 | 280 |
| 36 | West | Dhokung Dhara | 27.18 | 88.30 | 1210 | 420 |
| 37 | West | Karkharay Khola | 27.13 | 88.14 | 1587 | 390 |
| 38 | West | Singaney Source | 27.31 | 88.11 | 2137 | 270 |
| 39 | West | Dhara Pani | 27.14 | 88.06 | 2134 | 400 |
| 40 | West | Linkhim | 27.06 | 88.11 | 1592 | 600 |
