# Supplementary material for: Physicochemical Parameters and Alarming Coliform Count of the Potable Water of Eastern Himalayan State Sikkim: An Indication of Severe Fecal Contamination and Immediate Health Risk
Source: Front Public Health. 2019 Jul 10;7:174. doi: 10.3389/fpubh.2019.00174 (PMC6636254; doi:10.3389/fpubh.2019.00174)
Supplement: Supplementary Table S3 — Structured questionnaire. [file Table_3.DOCX]

**Risk Factor associated with Water, Sanitation and Hygiene**

| Q. 1 Demographics |
| --- |
| 1. Gender- Male/Female |
| 1. Age (yrs.)-(15-30)/ (31-45)/(46-60)/(61-75) |
| 1. Population type- a. Rural b. Semi-rural |
| 1. Name of Place------------- |
| 1. Name of Spring------------ |
| 1. District- North/west/East/West |

| Q. 2 General information  Frequency of diet intake-   1. Twice b. Thrice |
| --- |
| Use of Antibiotics-   1. Never b. Occasional c. Regular |
| Major Dietary composition-   1. Vegetarian b. Non-vegetarian c. Vegan |
| Diet Type-   1. Cocked b. Un-cooked c. Boiled d. Steamed e. Canned |
| Food Source-   1. Own Farm b. Market c. Both |
| Consumption of Fermented Food-   1. Yes b. No |
| Fermented Food type-   1. Gundaruk b. Kinema c. Churpi d. Dahi e. Dry Meat |
|  |

| Q. 3 Family Type:   1. Joint b. Nuclear |
| --- |
| Economic Status:   1. Below Poverty Line 2. Above Poverty Line |
| Cast:   1. Schedule Cast 2. Schedule Tribe 3. Others |
| Number of Family Members:   1. 5 member or less 2. 6 or more than 6   Paternal Education:   1. Matric 2. Higher Secondary 3. Graduate |
| Maternal Education:   1. Matric 2. Higher Secondary 3. Graduate |
| Paternal Occupation:   1. Agriculture work 2. Government Job 3. Other Work |
| Maternal Occupation:   1. Housework only 2. Housework + Government Job 3. Other Work |
| Antibiotic Used last Month:   1. Yes 2. No |

| Q. 4 Water Source-   1. Private 2. Natural Springs 3. Government supply 4. River   Q. 5 How much water you have stored for drinking:   1. 10liter 2. 20liter 3. 30liter 4. More than 50 liter   Q. 6 Is raw water is drinkable:   1. Yes 2. No   Q. 7 Treatment of Stored Water Before Drinking:  a. Yes  b. No  Q. 8 How often Storage tank disinfected:   1. Weekly 2. Monthly 3. Yearly 4. Not applicable   Q. 9 How your raw water transported:   1. Plastic Pipeline 2. Iron Pipeline 3. Tanker supply 4. Not known   Q. 10 Do you have separate water storage Place:   1. Yes 2. No   Q. 11 How often family members wash their hands (with soap or water) before taking water from storage:   1. Always 2. Often 3. Sometimes 4. Never   Q. 12 How you normally get water out of your container when you want to drink:   1. Directly with an unwashed hand 2. Directly, but after washing your hand 3. Using utensils 4. Tap   Q. 13 How many people normally live in your house:   1. Single 2. Three 3. 5 or less than 5 4. 6 or more than 6   Q. 14 How many members of the family are less than 6 years:   1. No one 2. 1 3. 2 4. More than 2   Q. 15 How often family members wash their hands with soap after toilet:   1. Always 2. Sometimes 3. Often 4. Never |
| --- |
| Q. 16 Water Consumed-   1. Raw 2. Boiled 3. Filtered 4. Purified   Q. 17 Are your water reservoir covered properly:   1. Yes 2. No   Q. 18Are your water reservoir protected by the reach of children:   1. Yes 2. No   Q. 19 Are your water reservoir has a different place from Smoking and Drinking area:   1. Yes 2. No |
| Q. 20 Quality of Water-   1. Outstanding 2. Good 3. Some chronic issue 4. Poor |
|  |
| Q. 21 How much water (say X liters) you drink in a day-   1. X=0 2. 0<X<1 3. 1<X<3 4. X>6 |
| Q. 22 Does the region suffer any type of waterborne diseases during a year-   1. Yes 2. No |
| Q. 23If Yes, then which type-   1. Diarrhea with vomiting 2. Diarrhea with fever 3. Diarrhea with blood 4. Only diarrhea 5. Abdominal pain and vomiting after consumption of food |
| Q. 24 Are you aware of water-related health diseases-   1. Yes 2. No   If Yes, Mentioned Name: |
| Q. 25 Did you or any of your family members ever have water-related health problems-   1. Yes 2. No   If Yes, Mention the name: |
| Q. 26 Which season is more prone to evoke water-borne diseases-   1. Rainy 2. Winter 3. Summer 4. Springs |
|  |
| Q. 27 How often the storage space for the supply water is cleaned in a Year-   1. Daily 2. Weekly 3. Monthly 4. Yearly 5. None of above |
| Q. 28 Can the industry/sewage treatment plant waste contaminate the water in the nearby locality-   1. Yes 2. No 3. N/A |
| Q. 29 What type of Water storage:   1. Direct 2. Plastic storage tank 3. Underground septic tank |
| Q. 30 Properties of Water (Observation) |
| pH of water-   1. >7 2. <7 3. =7 |
| Temperature of Water   1. Cold 2. Mild 3. Warm |
| Odour of Water-   1. Pungent 2. Odour-less 3. Others |

| Q. 31 Social/life style behavior |
| --- |
| Smoking-   1. Yes 2. No |
| Alcohol consumption-   1. Yes 2. No |
| Frequency of alcohol consumption-   1. Daily 2. Weekly 3. Occasionally |
| Preferred alcoholic beverage-   1. Wine 2. Brandy 3. Beer 4. Rum 5. Whisky 6. Home brew |
| Appropriate sanitary condition-   1. Yes 2. No |
| Cooking infrastructure-   1. Hygienic 2. Un-hygienic |
