# Supplementary figures and images for: Physicochemical Parameters and Alarming Coliform Count of the Potable Water of Eastern Himalayan State Sikkim: An Indication of Severe Fecal Contamination and Immediate Health Risk
Source: Front Public Health. 2019 Jul 10;7:174. doi: 10.3389/fpubh.2019.00174 (PMC6636254; doi:10.3389/fpubh.2019.00174)

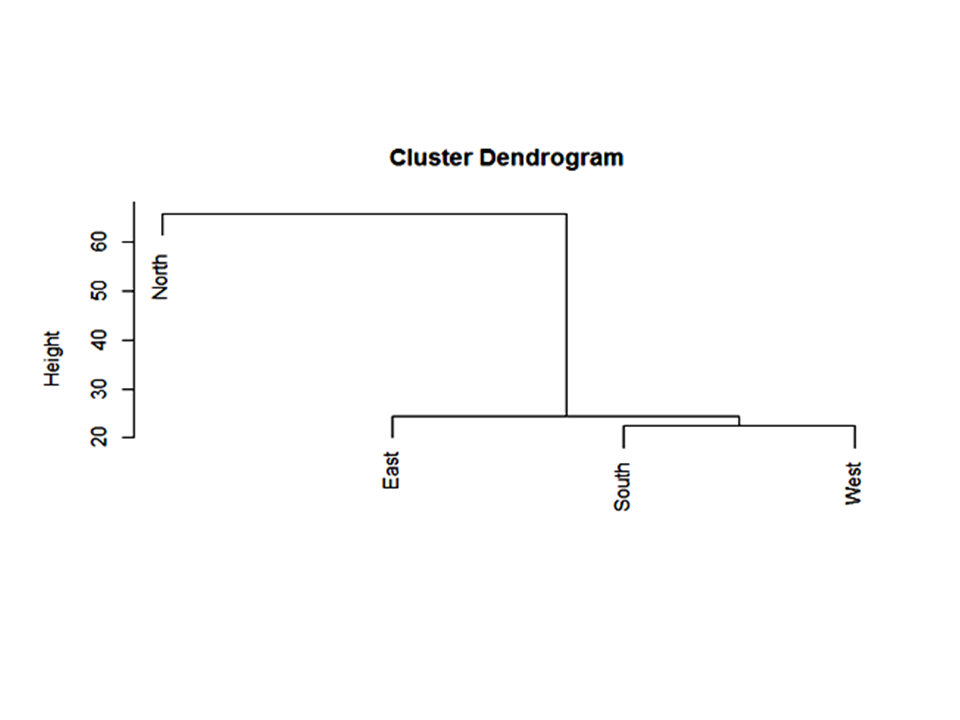

Supplement: Supplementary Figure S1 — Cluster analysis based on the mean concentration of total coliform, E. coli and Enterococcus. [file Image_1.TIF]

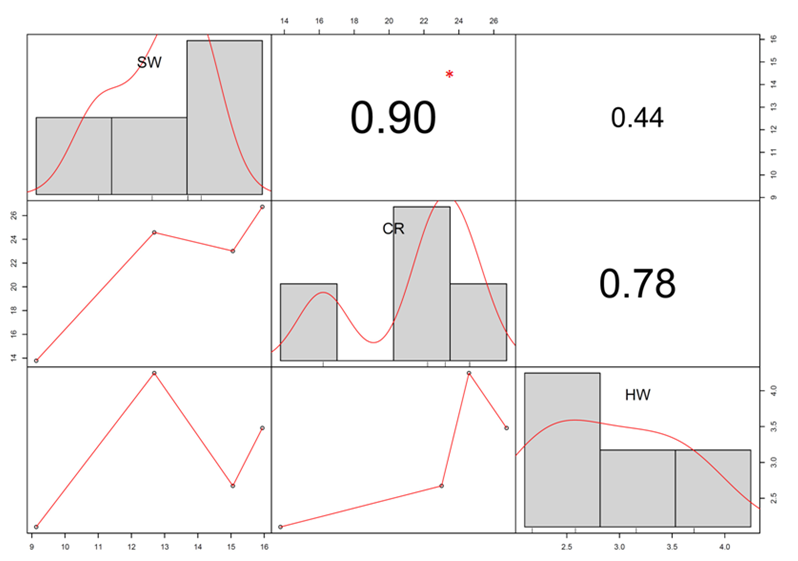

Supplement: Supplementary Figure S2 — The correlation between the total coliform count of spring water, community reservoir and household water. [file Image_2.TIF]
