# Supplementary material for: Physicochemical Parameters and Alarming Coliform Count of the Potable Water of Eastern Himalayan State Sikkim: An Indication of Severe Fecal Contamination and Immediate Health Risk
Source: Front Public Health. 2019 Jul 10;7:174. doi: 10.3389/fpubh.2019.00174 (PMC6636254; doi:10.3389/fpubh.2019.00174)
Supplement: Supplementary Data Sheet 1 — Report of several major waterborne diseases released by Ministry of Health and Family Welfare, Government of India. [file Data_Sheet_1.PDF]

GOVERNMENT OF INDIA
MINISTRY OF HEALTH AND FAMILY WELFARE
DEPARTMENT OF HEALTH AND FAMILY WELFARE

LOK SABHA
UNSTARRED QUESTION NO. 975
TO BE ANSWERED ON 21ST JULY, 2017

WATER BORNE DISEASES

975. SHRI NISHIKANT DUBEY:
SHRI RAVINDRA KUMAR PANDEY:
SHRI RAJESH PANDEY:

Will the Minister of **HEALTH AND FAMILY WELFARE** be pleased to state:

(a) whether a large number of people have contracted water borne diseases and if so, the details thereof over the last three years, State and UT-wise;

(b) the number of such cases that have been fatal;

(c) the steps the Government has taken to provide safe potable water in rural and urban areas across the country including Jharkhand; and

(d) the funds allocated and disbursed for the same during the last three years?

ANSWER
THE MINISTER OF STATE IN THE MINISTRY OF HEALTH AND FAMILY WELFARE
(SMT. ANUPRIYA PATEL)

(a) & (b): Diseases such as Cholera, Acute Diarrhoeal Diseases (Dysentery and Diarrhea), Enteric Fever (Typhoid) and Viral Hepatitis A & E are caused by consumption of contaminated drinking water. State/UT wise details of number of cases and deaths reported due to these diseases during 2014-16 as per data compiled by the Central Bureau of Health Intelligence (CBHI) are indicated in **Annexure.**

(c) & (d): Provision of safe drinking water and health services falls within the remit of the State/UT Governments. However, the Government of India has taken steps both for facilitating availability of safe drinking water and also for ensuring effective surveillance of outbreaks of such diseases and their prevention and management.

As informed by the Ministry of Drinking Water & Sanitation, all States have been informed to commission surface water based piped water supply schemes in all habitations as a long term sustainable solution. However, since these projects have a long gestation period (say 3-5 years) and the rural people cannot be put to the risk of consuming contaminated water, all States have also been advised to install community water purification plants. This has been done with the objective of providing 8-10 litres of safe water per capita per day for drinking and cooking purposes only.

Contd……

Besides, the Ministry of Health and Family Welfare, through its Integrated Disease Surveillance Programme, helps the States/UTs to effectively detect and respond to disease outbreaks including water borne diseases by providing additional manpower, strengthening of laboratories, Information & Communication Technology (ICT) equipment and funds. The National Centre for Disease Control (NCDC) also helps the States/UTs through surveillance, investigation, laboratory and other facilities. It also helps States in capacity building through training & developmental activities.

Health System Strengthening support being given to the State Governments under National Health Mission (NHM) also enhances capacity of the Health Institutions, up to the district level, to treat and manage water borne diseases.

As informed by Ministry of Drinking Water & Sanitation, funds are released and utilized for the supply of safe drinking water under National Rural Drinking Water Programme (NRDWP) including National Water Quality Sub Mission in the rural areas of the country. Besides, NITI Aayog has also provided funds to the tune of Rs. 800.00 crores during financial year 2015-16 for commissioning community water purification plants in all remaining arsenic and fluoride affected habitations as a short term/immediate measure. Utilization of resources optimally by the State Governments is critical for effectively reducing the incidence of water-borne diseases.

………………..

Annexure

State-wise Cases & Deaths Due to Cholera in India 2014 – 2016							
**Sl No**	**State / U.T.**	**2014**		**2015**		**2016 (Prov.)**	
		**Cases**	**Deaths**	**Cases**	**Deaths**	**Cases**	**Deaths**
1	Andhra Pradesh	0	0	0	0	0	0
2	Arunachal Pradesh	0	0	0	0	9	0
3	Assam	0	0	0	0	0	0
4	Bihar	0	0	0	0	0	0
5	Chhattisgarh	20	0	7	0	55	0
6	Goa	0	0	0	0	0	0
7	Gujarat	158	0	52	0	88	0
8	Haryana	7	0	0	0	0	0
9	Himachal Pradesh	0	0	0	0	0	0
10	J & K	0	0	0	0	0	0
11	Jharkhand	0	0	36	0	5	0
12	Karnataka	32	0	14	2	29	1
13	Kerala	3	0	0	0	7	0
14	Madhya Pradesh	17	0	104	0	94	1
15	Maharashtra	252	2	213	1	107	0
16	Manipur	0	0	0	0	0	0
17	Meghalaya	1	1	0	0	0	0
18	Mizoram	0	0	26	0	17	0
19	Nagaland	0	0	0	0	6	0
20	Odisha	0	0	2	0	0	0
21	Punjab	0	0	0	0	0	0
22	Rajasthan	56	0	3	0	2	0
23	Sikkim	0	0	0	0	0	0
24	Tamil Nadu	14	0	15	0	8	0
25	Telangana	NA	NA	0	0	0	0
26	Tripura	0	0	0	0	0	0
27	Uttarakhand	0	0	0	0	0	0
28	Uttar Pradesh	0	0	47	0	4	0
29	West Bengal	173	0	155	0	157	0
30	A & N Islands	0	0	0	0	0	0
31	Chandigarh	15	0	133	0	10	0
32	D & N Haveli	28	1	14	1	15	1
33	Daman & Diu	0	0	0	0	0	0
34	Delhi	65	1	91	0	228	0
35	Lakshadweep	0	0	0	0	0	0
36	Puducherry	3	0	1	0	0	0
**Total**		**844**	**5**	**913**	**4**	**841**	**3**

Source: National Health Profile brought out by CBHI, Dte.GHS

Note :

1. 2014: Andhra Pradesh Excludes data of 10 districts of Telangana from July 2014.
2. 2016: Data is different for different reference period.
3. NA stands for Not Available.

State-wise Cases and Deaths due to Acute Diarrhoeal Diseases reported during 2014 – 2016							
**Sl. No.**	**State / U.T.**	**2014**		**2015**		**2016 (Prov.)**	
		**Cases**	**Deaths**	**Cases**	**Deaths**	**Cases**	**Deaths**
1	Andhra Pradesh	1332145	10	1122740	5	1194005	11
2	Arunachal Pradesh	12657	5	10834	4	11715	2
3	Assam	83373	73	128392	121	88736	282
4	Bihar	550038	24	455125	36	392224	8
5	Chhattisgarh	115561	32	132278	13	157064	33
6	Goa	16097	4	13204	1	14245	4
7	Gujarat	504857	3	567123	3	641451	0
8	Haryana	197898	8	190390	4	224780	14
9	Himachal Pradesh	350459	52	334168	41	310789	56
10	J & K	515013	0	472843	2	534341	0
11	Jharkhand	81451	17	81934	0	93547	0
12	Karnataka	810781	12	832356	13	930369	4
13	Kerala	402106	9	428374	2	476686	15
14	Madhya Pradesh	768021	112	740690	74	740236	122
15	Maharashtra	664014	4	877638	27	1051445	52
16	Manipur	29954	32	29159	23	33193	21
17	Meghalaya	197024	29	167691	32	165404	29
18	Mizoram	14201	10	14215	11	13602	12
19	Nagaland	22301	0	15511	0	15062	0
20	Odisha	767575	190	782151	139	775824	103
21	Punjab	170438	22	179211	37	195281	44
22	Rajasthan	676832	17	810518	13	897209	7
23	Sikkim	39983	2	53295	3	46289	0
24	Tamil Nadu	250264	14	308358	8	367815	9
25	Telangana	NA	NA	963573	20	871497	17
26	Tripura	80388	22	88064	5	95278	6
27	Uttarakhand	90428	14	108974	6	110942	7
28	Uttar Pradesh	754582	301	814481	320	1066342	303
29	West Bengal	1896182	200	1798754	196	2045451	192
30	A & N Islands	23947	2	22398	0	23547	0
31	Chandigarh	39277	29	45284	90	49891	61
32	D & N Haveli	63337	0	51195	4	43280	12
33	Daman & Diu	12831	0	18169	2	13062	0
34	Delhi	120618	77	157445	96	135907	109
35	Lakshadweep	6750	0	4472	0	4387	0
36	Puducherry	87248	11	92599	2	92379	5
**Total**		**11748631**	**1337**	**12913606**	**1353**	**13923275**	**1540**

Source: National Health Profile brought out by CBHI, Dte.GHS

Note :

1. 2014: Andhra Pradesh Excludes data of 10 districts of Telangana from July 2014.
2. 2016: Data is different for different reference period.
3. NA stands for Not Available.

State-wise Cases and Deaths due to Enteric Fever (Typhoid) reported during 2014– 2016							
**Sl. No.**	**State / U.T.**	**2014**		**2015**		**2016 (Prov.)**	
		**Cases**	**Deaths**	**Cases**	**Deaths**	**Cases**	**Deaths**
1	Andhra Pradesh	186446	5	146385	0	170249	0
2	Arunachal Pradesh	4512	3	4476	10	4574	1
3	Assam	5328	29	11333	9	19328	0
4	Bihar	283679	4	265469	1	204366	2
5	Chhattisgarh	32617	1	47970	1	74632	8
6	Goa	573	0	1603	1	724	0
7	Gujarat	29505	0	35362	1	45970	0
8	Haryana	29990	1	31965	0	36442	0
9	Himachal Pradesh	48786	6	40639	6	38093	7
10	J & K	57537	1	52359	0	46904	0
11	Jharkhand	36663	7	28330	4	41731	0
12	Karnataka	92959	1	85837	1	97493	1
13	Kerala	2269	0	2862	0	2038	0
14	Madhya Pradesh	155190	25	125737	8	129998	21
15	Maharashtra	102299	0	130809	0	137617	0
16	Manipur	10636	10	5422	0	4942	2
17	Meghalaya	10395	8	13459	0	14128	0
18	Mizoram	2758	4	2804	0	3085	3
19	Nagaland	11604	0	7977	0	8267	0
20	Odisha	90363	39	90895	45	73330	18
21	Punjab	34651	1	34867	3	37896	3
22	Rajasthan	83540	4	79244	0	116470	0
23	Sikkim	716	0	453	2	474	0
24	Tamil Nadu	29937	0	40579	0	33853	0
25	Telangana	NA	NA	163747	0	133838	2
26	Tripura	10553	0	4596	1	5398	0
27	Uttarakhand	28939	14	34120	10	33904	4
28	Uttar Pradesh	225829	203	288140	221	495698	313
29	West Bengal	90086	42	112262	24	161264	22
30	A & N Islands	881	0	870	0	1127	1
31	Chandigarh	6021	0	12447	88	12237	48
32	D & N Haveli	2439	0	1406	0	4420	1
33	Daman & Diu	167	0	165	0	197	0
34	Delhi	27339	14	30698	16	30015	55
35	Lakshadweep	3	0	77	0	50	0
36	Puducherry	1477	3	2049	0	1943	0
Total		**1736687**	**425**	**1937413**	**452**	**2222695**	**512**

Source: National Health Profile brought out by CBHI, Dte.GHS

Note :

1. 2014: Andhra Pradesh Excludes data of 10 districts of Telangana from July 2014.
2. 2016: Data is different for different reference period.
3. NA stands for Not Available.

State-wise Cases and Deaths due to Viral Hepatitis (All Causes) reported during 2014 – 2016							
**Sl. No.**	**State / U.T.**	**2014**		**2015**		**2016 (Prov.)**	
		**Cases**	**Deaths**	**Cases**	**Deaths**	**Cases**	**Deaths**
1	Andhra Pradesh	3716	1	3358	12	2662	1
2	Arunachal Pradesh	378	6	292	1	271	4
3	Assam	2033	13	809	9	2688	14
4	Bihar	20670	3	26729	2	28578	0
5	Chhattisgarh	548	4	532	6	547	15
6	Goa	182	0	162	0	121	0
7	Gujarat	4808	7	3736	0	3573	2
8	Haryana	1934	13	5184	3	2274	0
9	Himachal Pradesh	2808	9	1739	1	2716	18
10	J & K	5110	0	4028	0	3581	0
11	Jharkhand	1052	1	1258	1	1406	0
12	Karnataka	6402	8	6026	21	6013	17
13	Kerala	5567	4	3965	7	5327	18
14	Madhya Pradesh	16145	18	14030	25	12158	22
15	Maharashtra	6753	13	9738	10	12156	6
16	Manipur	443	0	88	0	182	1
17	Meghalaya	643	0	299	0	236	2
18	Mizoram	194	0	209	4	276	1
19	Nagaland	113	0	76	0	86	0
20	Odisha	5069	39	5146	24	3481	19
21	Punjab	4525	4	9330	8	8833	7
22	Rajasthan	9719	3	3247	0	2471	1
23	Sikkim	556	0	1344	0	800	0
24	Tamil Nadu	880	0	1066	1	715	0
25	Telangana	NA	NA	1735	1	2175	0
26	Tripura	177	1	183	1	363	4
27	Uttarakhand	9243	9	10242	15	10930	16
28	Uttar Pradesh	16037	50	11188	73	12530	47
29	West Bengal	4444	63	3948	83	2898	115
30	A & N Islands	262	9	123	1	177	0
31	Chandigarh	766	21	1249	27	1179	7
32	D & N Haveli	32	0	48	6	7	2
33	Daman & Diu	65	0	77	2	31	0
34	Delhi	6965	98	9145	87	10281	100
35	Lakshadweep	16	0	12	0	10	0
36	Puducherry	299	3	520	4	416	7
Total		**138554**	**400**	**140861**	**435**	**142148**	**446**

Source: National Health Profile brought out by CBHI, Dte.GHS

Note :

1. 2014: Andhra Pradesh Excludes data of 10 districts of Telangana from July 2014.
2. 2016: Data is different for different reference period.
3. NA stands for Not Available.
